# Prevalence and properties of *mecC* methicillin-resistant *Staphylococcus aureus* (MRSA) in bovine bulk tank milk in Great Britain

**DOI:** 10.1093/jac/dkt417

**Published:** 2013-10-23

**Authors:** G. K. Paterson, F. J. E. Morgan, E. M. Harrison, S. J. Peacock, J. Parkhill, R. N. Zadoks, M. A. Holmes

**Affiliations:** 1 Department of Veterinary Medicine, University of Cambridge, Madingley Road, Cambridge CB3 0ES, UK; 2 Health Protection Agency, Microbiology Services Division Cambridge, Level 6 Addenbrooke's Hospital, Hills Road, Cambridge CB2 0QQ, UK; 3 Department of Medicine, University of Cambridge, Addenbrooke’s Hospital, Cambridge CB2 0QQ, UK; 4 Cambridge University Hospitals NHS Foundation Trust, Cambridge CB2 0QQ, UK; 5 The Wellcome Trust Sanger Institute, Wellcome Trust, Genome Campus, Cambridge CB10 1SA, UK; 6 Moredun Research Institute, Bush Loan, Penicuik EH26 0PZ, UK; 7 Institute of Biodiversity, Animal Health and Comparative Medicine, College of Medical, Veterinary and Life Sciences, University of Glasgow, 464 Bearsden Road, Glasgow G61 1QH, UK

**Keywords:** bovine mastitis, antibiotic resistance, molecular epidemiology

## Abstract

**Objectives:**

*mecC* methicillin-resistant *Staphylococcus aureus* (MRSA) represent a newly recognized form of MRSA, distinguished by the possession of a divergent *mecA* homologue, *mecC*. The first isolate to be identified came from bovine milk, but there are few data on the prevalence of *mecC* MRSA among dairy cattle. The aim of this study was to conduct a prevalence study of *mecC* MRSA among dairy farms in Great Britain.

**Methods:**

Test farms were randomly selected by random order generation and bulk tank samples were tested for the presence of *mecC* MRSA by broth enrichment and plating onto chromogenic agar. All MRSA isolated were screened by PCR for *mecA* and *mecC*, and *mecC* MRSA were further characterized by multilocus sequence typing, *spa* typing and antimicrobial susceptibility testing.

**Results:**

*mecC* MRSA were detected on 10 of 465 dairy farms sampled in England and Wales (prevalence 2.15%, 95% CI 1.17%–3.91%), but not from 625 farms sampled in Scotland (95% CI of prevalence 0%–0.61%). Seven isolates belonged to sequence type (ST) 425, while the other three belonged to clonal complex 130. Resistance to non-β-lactam antibiotics was uncommon. All 10 isolates produced a negative result by slide agglutination for penicillin-binding protein 2a. *mecA* MRSA ST398 was detected on one farm in England.

**Conclusions:**

*mecC* MRSA is widely distributed among dairy farms in Great Britain, but this distribution is not uniform across the whole country. These results provide an important baseline dataset to monitor the epidemiology of this emerging form of MRSA.

## Introduction

Methicillin-resistant *Staphylococcus aureus* (MRSA) encoding a divergent *mecA* homologue within a novel SCC*mec* type XI element were first reported in bovine and human isolates from the UK, Denmark and Eire in 2011.^1,2^ Originally named *mecA*_LGA251_, and subsequently designated *mecC*,^3^ this homologue shares 69% nucleotide identity with *mecA* and produces a negative result in *mecA*-based PCR assays and slide agglutination tests for penicillin-binding protein (PBP) 2a.

As a result of its recent discovery and these diagnostic difficulties, there are relatively few data on the prevalence and epidemiology of *mecC* MRSA. However, *mecC* MRSA isolates have now been reported from a number of additional European countries, including France,^4^ Finland,^5^ Sweden,^6^ the Netherlands,^7^ Germany,^7–9^ Austria,^10^ Switzerland,^11^ Norway^12^ and Belgium,^13,14^ and from a broad range of host species, encompassing livestock, companion animals and wildlife, including sheep,^13,15^ domestic cat,^12,16^ domestic dog,^13,16^ brown rat,^13^ hare,^10^ rabbit,^13^ otter,^10^ hedgehog,^6^ guinea pig,^16^ common seal^13^ and chaffinch.^13^ Furthermore, zoonotic transmission from livestock to humans in Denmark has been corroborated by epidemiological follow-up and whole genome sequencing.^17,18^ *mecC* has also been detected in other species of staphylococci, specifically *Staphylococcus xylosus* from bovine mastitis^19^ and *Staphylococcus stepanovicii* from a wild European lynx.^10^

Given that the original *mecC* MRSA isolate was found in bovine milk and that *S. aureus* is an important cause of bovine mastitis,^20^ we undertook a prevalence study of *mecC* MRSA in dairy bulk tank samples in Great Britain (GB) collected during 2011–12.

## Materials and methods

### Bulk tank milk samples and processing

Randomly selected bulk tank milk samples were supplied between November 2011 and October 2012 by National Milk Laboratories Ltd (Chippenham, UK), a commercial milk testing company responsible for >95% of quality assurance testing of bulk tank milk from GB dairy farms. The dairy farms to be sampled were selected by taking a list of all farms using National Milk Laboratories Ltd and placing these in random order using Microsoft Excel (Seattle, WA, USA), with the first 500 selected for sampling.

Samples from England and Wales were treated together as these are collected and processed by one laboratory, with Scottish samples processed at another. Of the 500 selected farms in England and Wales, 35 had ceased production of milk before they could be tested, and so were removed from the study, leaving a sample size of 465 dairy farms. In Scotland, the same process was used except the sampling continued beyond the first 500 farms when no *mecC* MRSA-positive farms were discovered in any of these, enlarging the final sample size to 625 dairy farms to increase the confidence limits of the result.

The bulk tank milk samples were collected aseptically by trained technicians for quality assurance purposes and stored at 4°C for up to 5days before freezing at −20°C prior to testing. Samples were thawed at 37°C and 1 mL of milk was added to 4 mL of Mueller–Hinton broth (Oxoid, Basingstoke, UK) supplemented with 6.5% (w/v) NaCl. After incubation at 37°C with shaking at 200 rpm, 50 μL of culture was spread onto MRSA Brilliance 2 plates (Oxoid) and incubated at 37°C for 24 h. Experiments with spiked milk indicated a lower limit of detection for *mecC* MRSA of ≤50 cfu/mL of milk. Potential MRSA colonies (blue colour) were subcultured on Staph Brilliance 24 plates (Oxoid) and subsequently screened for *mecA*, *mecC* and *femB* by multiplex PCR as described previously.^13^ All *mecC* MRSA isolates were genome sequenced to confirm them as *mecC* positive, and the multilocus sequence type (ST) was derived from the genome sequence.

### Antibiotic susceptibility

Resistance to oxacillin, cefoxitin, chloramphenicol, ciprofloxacin, tetracycline, erythromycin, mupirocin, clindamycin, trimethoprim, gentamicin, linezolid, penicillin, fusidic acid, neomycin and rifampicin was assessed by disc diffusion (Oxoid) following BSAC guidelines (version 11.1 2012) and using NCTC 6571 and NCTC 12493 as control strains. In addition, the MICs of oxacillin and cefoxitin were determined using the Etest (bioMérieux, Basingstoke, UK).

### Slide agglutination for PBP2a

Bovine *mecC* MRSA isolates were tested with three commercially available PBP2a detection assays: the Mastalex™ MRSA Test (MAST, Bootle, UK), the Penicillin Binding Protein (PBP2′) Latex Agglutination Test (Oxoid) and the Alere™ PBP2a Culture Colony Test, according to the manufacturers' instructions. The *mecA*-positive strain NCTC 12493 was used as a positive control.

## Results

### Prevalence of mecC MRSA among GB dairy farm bulk milk

**Figure 1.**
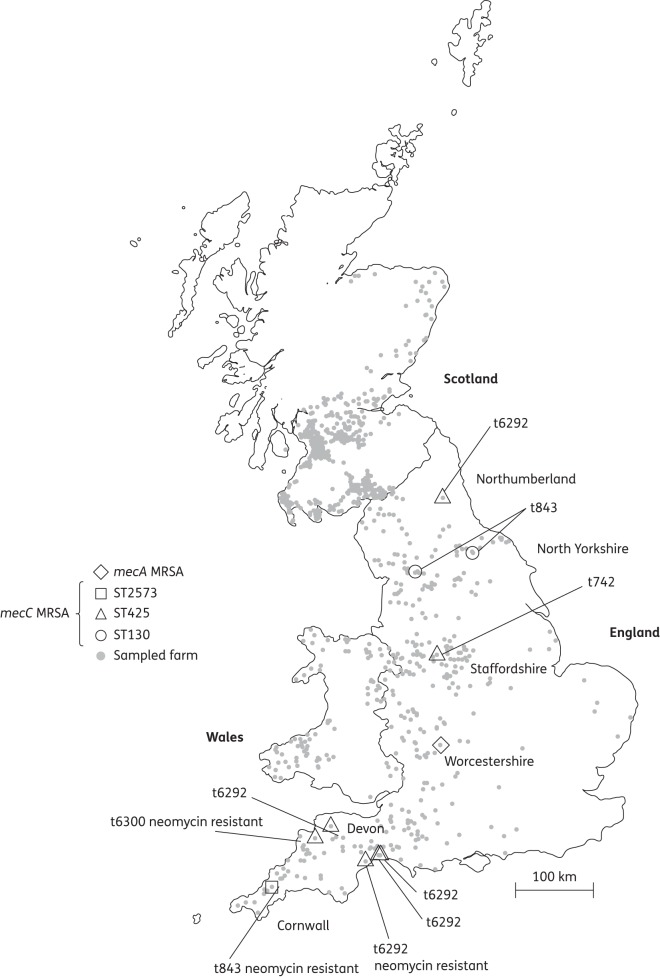
Map of GB dairy farms sampled for MRSA. All sampled farms are shown; 1079 farms were MRSA negative, 10 farms were *mecC* MRSA positive and 1 farm was *mecA* MRSA positive. Multilocus ST, *spa* type and additional antibiotic resistance are indicated for *mecC* MRSA.

From England and Wales, 10 dairy farms from a total of 465 sampled farms were positive for *mecC* MRSA (Figure 1). This represents a prevalence rate of 2.15% (95% CI 1.17%–3.91%). None of the positive farms was in Wales (*n* = 90), giving a prevalence of 0% (95% CI 0%–4.09%), and providing a prevalence of 2.67% (95% CI 1.46%–4.84%) for the 375 dairy farms sampled in England. *mecC* MRSA-positive farms were found in five counties distributed from the south-west (Devon and Cornwall) to the north of England (Northumberland) (Figure 1). None of the original 500 samples from Scotland was positive for *mecC* MRSA and so an additional 125 samples were tested. None of these 625 Scottish samples was positive for *mecC* MRSA (95% CI 0%–0.61%). A comparison of the England and Wales prevalence with the Scotland prevalence using Fisher's exact test rejected the null hypothesis of ‘no difference between the two rates’ with *P* = 0.0002.

### Characterization of bovine mecC MRSA

Seven of the ten *mecC* MRSA isolates belonged to ST425, among which three *spa* types were represented (t6292, t742 and t6300). The three remaining isolates belonged to clonal complex (CC) 130, two of which were ST130 and the third was ST2573, a novel *yqiL* single locus variant of ST130. All three CC130 isolates belonged to *spa* type t843. All 10 isolates were resistant to cefoxitin by disc diffusion with the Etest showing MICs varying from 6 mg/L to 12 mg/L. The MIC of oxacillin was more variable, ranging from 6 mg/L to 48 mg/L, and all isolates were also resistant to oxacillin as determined using disc diffusion.

All 10 strains produced a negative result using the three commercially available PBP2a detection assays. Strains were also tested for susceptibility to chloramphenicol, ciprofloxacin, tetracycline, erythromycin, mupirocin, clindamycin, trimethoprim, gentamicin, linezolid, penicillin, fusidic acid, neomycin and rifampicin. While all strains were resistant to penicillin, as might have been expected, resistance to non-β-lactams was uncommon, with three strains being resistant to neomycin as the only other resistance seen (Figure 1).

### Prevalence and characteristics of mecA MRSA from GB bulk milk

In addition to being screened for *mecC*, putative MRSA from the prevalence study samples were also screened by PCR for *mecA*. This identified a single farm in Worcestershire, England that was positive for *mecA* MRSA (Figure 1) and gives a prevalence in England of 0.27% (95% CI 0.05%–1.50%). This isolate belonged to ST398.

## Discussion

Here we report the first prevalence study of *mecC* MRSA on GB dairy farms. *mecC* MRSA was present on 10 farms of 465 in England and Wales, but was not present on 625 farms sampled in Scotland. The absence of previous data and the recent increase in *mecC* MRSA isolation from humans in Denmark^17^ indicate the importance of monitoring of *mecC* MRSA prevalence. The data reported here provide a valuable baseline for the future surveillance of emergent *mecC* MRSA and show that it is already widespread among the English dairy herd.

Although most *mecC* MRSA-positive farms were in the south-west of England, positive farms were also recorded elsewhere, indicating that *mecC* MRSA among English dairy farms is not localized to one particular region. Nevertheless, a degree of regional clustering of STs and *spa* types was observed, e.g. both isolates in North Yorkshire were ST130, t843 and four of five isolates in Devon were ST425, t6292. The cause of such patterns is not yet clear and is under investigation, but may be the result of local cattle movements and/or shared local services.

The difference in prevalence between Scotland and England is statistically significant, although the reasons for this are not obvious, especially as *mecC* MRSA has been isolated from other host species in Scotland – including humans and wildlife.^1,13^ While no survey samples from Wales were positive for *mecC,* MRSA testing of additional milk samples that were not part of the formal prevalence study found two dairy farms in Wales (Wrexham and Gwynedd) positive for *mecC* MRSA (data not shown).

The majority of bovine isolates found in this study belonged to ST425, with the others belonging to CC130, including ST2573 – a novel single locus variant of ST130. Although *mecC* MRSA has been isolated from bovine mastitis previously,^1,21^ the current study examined bulk tank milk samples and so the association of these isolates with clinical disease is unclear. Interestingly, of the *mecC* MRSA isolates identified by Garcia-Alvarez *et al*.^1^ from clinical bovine mastitis, the majority (11/13) belonged to CC130, in contrast to the majority of isolates from bulk tank milk samples in the present study (7/10), which belonged to ST425, as did the first *mecC* MRSA isolated by Garcia-Alvarez *et al*.^1^ This observation may suggest that there are differences in the propensity of *mecC* MRSA lineages to cause clinical bovine mastitis, with CC130 more likely to cause clinical disease than ST425. Indeed, an association between colonization site and ST has been demonstrated previously for certain bovine *S. aureus* STs,^22^ and ST425 may be colonizing the teat skin rather than tissue within the mammary gland. A small number of *mecC* MRSA isolates have been isolated from dairy cattle in Belgium,^14^ Denmark^17^ and Sweden,^23^ and belonged to ST130 (four isolates), ST425 (one isolate) and ST2508 (one isolate). Similar to human *mecC* MRSA isolates, it appears therefore that CC130 and ST425 are also the predominant *mecC* MRSA lineages in dairy cattle.^1^

The presence of *mecC* MRSA in dairy cattle may represent a zoonotic risk to humans, and two case studies using traditional epidemiology and genome sequencing have identified possible zoonotic transmission between livestock and humans.^17,18^ To assess this risk we conducted a prevalence survey of *mecC* MRSA nasal colonization among delegates at a British cattle veterinarian conference in 2011.^24^ However, none was positive for *mecC* MRSA, suggesting a prevalence of <1% in this population based on the sample size.^24^ An investigation of the phylogenetic relationships between the human and bovine isolates described here and previously^1^ is currently underway using whole genome sequencing.

Based on disc diffusion and MIC determination by the Etest, all 10 isolates were resistant to both cefoxitin and oxacillin and therefore were phenotypically MRSA. However, all 10 strains produced a negative result using three different commercially available PBP2a assays. Variable results using these assays have been reported using human isolates^1,2,8^ and our data show that these assays are not likely to be useful for the diagnosis of bovine *mecC* MRSA strains either.

In addition to *mecC* MRSA, the multiplex PCR also allowed the identification of *mecA* MRSA, and a single *mecA* MRSA-positive dairy farm was identified. The prevalence rates reported here suggests *mecA* MRSA is considerably less frequent on GB dairy farms than *mecC* MRSA. This *mecA* isolate belonged to ST398 and, along with bulk tank ST398 isolates from four other UK dairy farms, was reported previously as the first detection of livestock-associated CC398 MRSA in the UK dairy herd.^25^

We have presented the first prevalence study of *mecC* MRSA on GB dairy farms. These data provide a valuable baseline dataset for the future surveillance of this emerging veterinary and human pathogen.

## Funding

This work was supported by a Medical Research Council Partnership Grant (G1001787/1) held between the Department of Veterinary Medicine, University of Cambridge (M. A. H.), the School of Clinical Medicine, University of Cambridge (S. J. P.), the Moredun Research Institute (R. N. Z.) and the Wellcome Trust Sanger Institute (J. P. and S. J. P.).

## Transparency declarations

Competing interests: none to declare.

The funder had no role in the study design, data collection, analysis, decision to publish, or preparation of the manuscript.
